# The Genetic Structure of an Invasive Pest, the Asian Citrus Psyllid *Diaphorina citri* (Hemiptera: Liviidae)

**DOI:** 10.1371/journal.pone.0115749

**Published:** 2014-12-29

**Authors:** Aline S. Guidolin, Pablo Fresia, Fernando L. Cônsoli

**Affiliations:** 1 Lab de Interações em Insetos, Depto de Entomologia & Acarologia, ESALQ, Univ de São Paulo, Av. Pádua Dias 11, 13418-900, Piracicaba, São Paulo, Brasil; 2 Lab de Resistência de Artrópodes a Táticas de Controle, Depto de Entomologia & Acarologia, ESALQ, Univ de São Paulo, Av. Pádua Dias 11, 13418-900, Piracicaba, São Paulo, Brasil; Naval Research Laboratory, United States of America

## Abstract

The Asian citrus psyllid *Diaphorina citri* is currently the major threat to the citrus industry as it is the vector of *Candidatus* Liberibacter, the causal agent of huanglongbing disease (HLB). *D. citri* is native to Asia and now colonizes the Americas. Although it has been known in some countries for a long time, invasion routes remain undetermined. There are no efficient control methods for the HLB despite the intensive management tools currently in use. We investigated the genetic variability and structure of populations of *D. citri* to aid in the decision making processes toward sustainable management of this species/disease. We employed different methods to quantify and compare the genetic diversity and structure of *D. citri* populations among 36 localities in Brazil, using an almost complete sequence of the cytochrome oxidase I (COI) gene. Our analyses led to the identification of two geographically and genetically structured groups. The indices of molecular diversity pointed to a recent population expansion, and we discuss the role of multiple invasion events in this scenario. We also argue that such genetic diversity and population structure may have implications for the best management strategies to be adopted for controlling this psyllid and/or the disease it vectors in Brazil.

## Introduction

Biological invasions are of growing concern as they negatively impact agriculture and food security, ecosystem functioning, human health and the well-being in invaded areas. The analysis of invasive pests in newly invaded areas often needs to deal with the ignorance of species sources and/or invasion routes, information that can be fundamental to address practical aspects for managing invasive species [1]. Moreover, some low frequency states of biological traits of invasive species can be favored by selection. Anthropogenic activity must be considered in addition to the natural changes an invasive species suffers, since genetic diversity is influenced by management strategies and transport systems. Understanding the dynamics of this complex scenario is a difficult task and its impact on the genetic variability and population structure is currently under intense debate [2], [3].

Population structure can have a major impact on the conservation of endangered species and on pest management programs [4], [5]. In such a situation, selection may favour one phenotype over others. As a result, subpopulations can develop different ecological traits, such as host preference, mating preference, development time, pathogenicity, vector capacity, susceptibility to natural enemies and pesticides, and tolerance to heat, and other abiotic stressors [6], [7], [8], [9].

Mitochondrial DNA analysis has long been used for phylogenetic and evolutionary inferences due to its unique genetic and structural characteristics that allow for the identification of individuals or groups within a species [10], [11], [12], [13], [14], [15], [16], [17]. Furthermore, mutations in the cytochrome oxidase gene can result in a number of illnesses [18], [19], [20] or changes in life-history traits that influence insect fitness (development, lifespan, resistance to abiotic stress) [21], [22], [23].

The Asian citrus psyllid *Diaphorina* is a key pest in major citrus-growing countries as it vectors the bacteria *Candidatus* Liberibacter asiaticus and/or *Ca.* L. americanus, causal agents of the “huanglongbing” (HLB) [24], [25]. *D. citri* is a small insect that feeds on the phloem of young leaves and stems as immature, but also on older leaves of their host plants as adult by using its piercing-sucking mouthparts [26], [27], [28]. The development time, survival and reproduction of *D. citri* are severely affected by host plants and abiotic factors [29], [30], [31]. Population dynamics strongly correlate with the growth of young flushes on their host plants as these are the substrate to stimulate egg laying in *D. citri* females [27]. Young flushes are also the substrate used by nymphs to feed on [27]. *D. citri* was first described from Taiwan in 1907 [26]. It is currently known to occur throughout Asia, in the Middle East, the southern part of the United States of America, Caribbean islands, Central and South America [26], [27].

In Brazil, *D. citri* was first reported in the late 1930 s [32], but received little attention and was not a target insect for pest management until HLB was first detected in 2004 [24]. The inattention coupled with confounding patterns generated by the anthropogenic activity limit our present understanding of the demographic history of this invasive pest. Therefore, we aimed to assess population structure and thus infer the demographic history of *D. citri* by studying the genetic variability of 36 populations in the major citrus growing regions in Brazil. These data would allow a better understanding of the diversity of phenotypes and their involvement in the spread of the disease and resilience to the management tools in practice. We report *D. citri* populations form two geographically and genetically structured groups and have gone to a recent population expansion. The importance of such information in the adoption of management strategies to control the vector and the spread of the disease is discussed.

## Materials and Methods

### Insects

Adult samples of *D. citri* were collected from 36 localities in different regions of Brazil from 2005 to 2010, 32 of which from the state of São Paulo, four from the states of Roraima, Mato Grosso do Sul, Minas Gerais and Paraná (see Table 1 for sample location and coordinates). All samples were collected in commercial citrus orchards or in non-commercial private land with the consent of the land owners, and did not involve any endangered and/or protected conservation areas or species. Field collections in the state of São Paulo, Mato Grosso and Minas Gerais were carried out by field specialists of the “Fundo de Defesa da Citricultura” (Fundecitrus – www.fundecitrus.com.br), a private association of citrus growers and citrus industry, and sampled areas were owned by associated citrus growers or citrus industries. The sample from Paraná was kindly provided by Dr. Fábio A.C. Dossi (ESALQ/USP), and the sample obtained from Roraima was provided by Dr. Alberto L. Marsaro Jr. (EMBRAPA-Roraima). Each sample consisted of at least 10 insects collected from new shoots of different plants of citrus or orange jasmine (*Murraya paniculata*) in order to avoid the sampling of siblings. The collected specimens were immediately fixed in absolute ethanol for subsequent genomic DNA extraction.

**Table 1:** Group, localities, coordinates, host plant, number of individuals analysed (N), haplotypes, and nucleotide and haplotype diversity of *Diaphorina citri* in each sampled locality in Brazil.

Group	ID	Localities	Latitude	Longitude	CollectionDate	HostPlant	N	Haplotype(numberof individuals)	Nucleotidediversity ±SD	Haplotypediversity ±SD
I	1	Araraquara	21°47′S	48°10′W	01.iii.2005	Orangejasmine	7	H1(5), H13(1),H20(1)	0.000±0.000	0.523±0.028
	2	Barretos	20°33′S	48°34′W	27.x.2009	Orangejasmine	8	H1(3), H2(1), H7(1),H11(2), H23(1)	0.002±0.001	0.857±0.108
	3	Bebedouro	20°56′S	48°28′W	20.viii.2010	Orangejasmine	1	H1(1)	0.000±0.000	1.000±0.000
	4	Botucatu	22°53′S	48°26′W	01.iii.2005	Citrus	4	H3(2), H5(2)	0.001±0.001	0.666±0.204
	5	Cafelândia	21°48′S	49°36′W	01.iii.2005	Citrus	4	H1(3), H5(1)	0.001±0.001	0.500±0.265
	6	Casa Branca	21°46′S	47°05′W	01.iii.2005	Citrus	4	H1(2), H16(1), H44(1)	0.005±0.003	0.833±0.222
	7	Cerqueira César	23°02′S	49°09′W	10.x.2008	Citrus	6	H1(3), H2(1),H17(1), H18(1)	0.001±0.000	0.800±0.172
	8	Espírito Santodo Turvo	22°41′S	49°25′W	15.ix.2008	Citrus	9	H1(4), H2(1), H12(1),H19(1), H35(2)	0.003±0.002	0.833±0.126
	9	Guarantã	21°53′S	49°35′W	01.iii.2005	Citrus	5	H1(1), H11(1), H27(1),H33(1), H39(1)	0.004±0.003	1.000±0.126
	10	Itapetininga	23°35′S	48°03′W	01. xii.2008	Citrus	7	H1(5), H5(1), H41(1)	0.001±0.001	0.523±0.208
	11	Itirapina	22°15′S	47°49′W	01.iii.2005	Citrus	2	H3(1), H13(1)	0.002±0.002	1.000±0.500
	12	Mogi Mirim	22°25′S	46°57′W	21.viii.2010	Orangejasmine	5	H1(1), H28(1),H34(2), H38(1)	0.003±0.002	1.000±0.126
	13	Monte Alto	21°15′S	48°29′W	17.x.2009	Orangejasmine	4	H1(1), H3(1),H9(1), H15(1)	0.002±0.001	1.000±0.176
	14	Nova Granada	20°32′S	49°18′W	23.xi.2009	Citrus	7	H1(1), H3(2), H24(1),H26(1), H36(1), H40(1)	0.003±0.002	0.952±0.095
	15	Novo Horizonte	21°28′S	49°13′W	05.xi.2009	Citrus	6	H1(1), H3(2), H7(1),H10(1), H47(1)	0.005±0.003	0.933±0.121
	16	Penápolis	21°25′S	50°04′W	02.xii.2009	Orangejasmine	9	H6(1), H7(1), H9(1),H10(3), H31(1)H42(1), 45(1)	0.006±0.003	0.972±0.064
	17	Piracicaba	22°43′S	47°38′W	19.xi.2008	Orangejasmine	5	H1(3), H9(1), H11(1)	0.001±0.001	0.700±0.218
	18	Pongai	21°44′S	49°22′W	01.iii.2005	Citrus	2	H41(2)	0.002±0.002	1.000±0.500
	19	Potirendaba	21°02′S	49°22′W	01.xii.2009	Orangejasmine	5	H3(2), H16(1),H37(1), H43(1)	0.007±0.004	0.900±0.171
	20	Regianópolis	21°53′S	49°13′W	26.ix.2008	Citrus	6	H1(5), H2(1)	0.000±0.000	0.333±0.215
	21	Ribeirão Preto	21°10′S	47°48′W	24.xii.2008	Orangejasmine	6	H1(6)	0.000±0.000	0.000±0.000
	22	Sales	24°01′S	47°54′W	22.x.2009	Citrus	3	H1(1), H6(1), H32(1)	0.004±0.003	1.000±0.272
	23	Santa Cruz daConceição	22°08′S	47°27′W	02.xii.2009	Citrus	6	H1(1), H3(2), H4(1),H5(1), H9(1)	0.001±0.001	0.933±0.121
	24	São Carlos	22°01′S	47°53′W	01.iii.2005	Citrus	5	H1(5)	0.000±0.000	0.000±0.000
	25	Urupês	21°12′S	49°17′W	21.x.2009	Citrus	7	H1(6), H2(1)	0.000±0.000	0.285±0.196
II	26	BandeirantesD’Oeste	20°36′S	50°48′W	23.x.2008	Orangejasmine	4	H2(2), H6(1), H22(1)	0.001±0.000	0.900±0.161
	27	Cardoso	20°04′S	49°54′W	28.x.2009	Orangejasmine	7	H2(2), H4(1), H7(2),H8(1), H29(1)	0.002±0.001	0.866±0.129
	28	Palestina	20°23′S	49°25′W	11.xii.2009	Orangejasmine	8	H2(1), H4(2), H6(2),H8(1), H14(1), H15(1)	0.001±0.001	0.928±0.084
	29	Palmeira D’Oeste	20°24′S	50°45′W	07.x.2009	Orangejasmine	8	H1(1), H2(6), H6(1)	0.000±0.000	0.464±0.200
	30	Santa Fé do Sul	20°12′S	50°55′W	01.iii.2005	Citrus	8	H2(6), H8(1), H21(1)	0.000±0.000	0.464±0.200
	31	SudMenucci	20°37′S	50°52′W	23.x.2008	Orangejasmine	5	H2(1), H4(1),H5(2), H8(1)	0.002±0.001	0.900±0.161
	32	Três Fronteiras	20°14′S	50°53′W	16.x.2009	Orangejasmine	4	H2(1), H3(1),H10(1), H14(1)	0.01±0.001	1.000±0.176
	33	Três Lagoas – MS	20°45′S	51°40′W	31.i.2010	Orangejasmine	3	H2(2), H10(1)	0.000±0.000	0.666±0.314
	34	Divinópolis - MG	20°08′S	44°53′W	31.x.2010	Citrus	2	H2(1), H4(1)	0.003±0.003	1.000±0.500
	35	Maringá - PR	23°25′S	51°56′W	20.iv.2009	Citrus	4	H2(2), H4(1), H30(1)	0.002±0.001	0.833±0.222
	*36	Boa Vista – RR	2°49′N	60°40′W	19.vi.2009	Orangejasmine	8	H1(7), H25(1)	0.000±0.000	0.250±0.180

*Not assigned to a group.

### DNA extraction

Specimens were removed from ethanol, air dried at room temperature, and individually macerated in 100 µL of extraction buffer containing 10 mM tris(hydroxymethyl)aminomethane (Tris-HCl) at pH 8.0, 2 mM ethylenediaminetetraacetic acid (EDTA) at pH 8.0, 400 mM NaCl, 40 µL 20% sodium dodecyl sulphate and 8 µL proteinase K (20 mg·mL^−1^). Macerated samples were incubated at 55°C for 1 h, 300 µL of saturated aqueous NaCl solution (5 M) were added, and samples were vortexed (30 s) and centrifuged (14,000 *g*×30 min×25°C) for supernatant collection. Samples were mixed with 1 volume of cold isopropanol and incubated at −20°C overnight before centrifugation (14,000 *g*×20 min×4°C) [33]. The pelleted DNA was washed with ethanol, air dried, resuspended in 20 µL of autoclaved Milli-Q water, and stored at −20°C until further analysis. DNA integrity was assessed by electrophoresis on a 1% agarose gel slab containing 0.5 µg/mL ethidium bromide in Tris-acetate-EDTA buffer (40 mM Tris-acetate, 1 mM EDTA; pH 7.2) at 5 V/cm and visualized on a UV transilluminator.

### Cytochrome oxidase I amplification and sequencing

The genetic variability of *D. citri* was evaluated by amplifying a region of the cytochrome oxidase I (*COI*) gene. The 3′-end was amplified using the primer set DCITRI COI-L (5′AGGAGGTGGAGACCCAATCT-3′) and DCITRI COI-R (5′-TCAATTGGGGGAGAGTTTTG-3′) [34]. This sequence was extended by using new primers we developed to target the 5′-end: DcCOIF-ag (5′-CAATTG TAACTGCACACGCT-3′) or DcCOIF-agdeg (5′-CAATTGTAACWGCWCAYGCT-3′) and DcCOIR-ag (5′-GCTCGTGAGTCTACATCTAT-3′). All PCR reactions were carried out independently. Reactions were run with the following cycling parameters: 92°C for 5 min, 35 cycles at 92°C for 1 min, 53°C for 1 min, and 72°C for 1.5 min, and a final extension at 70°C for 10 min. Reactions were performed in a total volume of 25 µL containing 1–10 ng genomic DNA, 1× polymerase chain reaction (PCR) buffer, 1.5 mM MgCl_2_, 200 µM of each dNTP, 0.5 U Taq polymerase, and 0.32 µmol of each primer.

Amplicons were visualized on a UV transilluminator after electrophoresis on a 1.5% agarose gel slab containing 0.5 µg/mL ethidium bromide in TAE buffer. Samples were purified with ExoSAP (Fermentas) following the manufacturer’s guidelines. Amplified regions were subjected to bidirectional sequencing with primers from the original PCR reactions on an ABI 3700 automatic sequencer (Applied Biosystems, Foster City, CA), using the ABI Prism BigDye Kit protocol.

Chromatograms were visualized with FinchTV v.1.4.0 (Geospiza Inc.) and aligned using default parameters with the ClustalW algorithm as implemented in the MEGA v.5.05 software [35]. Sequence quality was evaluated by considering Phred values (threshold ≥20) and the final COI sequence was assembled by joining both partial sequences, the one targeting the 3′-end with the other targeting the 5′-end, using the tools available in the MEGA v.5.05 software [35]. The protein coding sequence was checked for the open reading frame by using the MEGA v.5.05 software [35].

### Genetic variability and differentiation

A total of 202 sequences of the mtDNA of *D. citri* from Brazil were included in the analyses. Haplotypes were assigned based on their nucleotide differences and their frequencies were obtained using the TCS v.1.21 [36]. Nucleotide (π) and haplotype (H^) diversities were estimated as defined by Nei [37] using the software Arlequin v.3.5.1.2 [38]. A haplotype network was inferred using the software TCS v.1.21 [36] with 95% as a connection limit, and modified following Crandall and Templeton [39]. The network was illustrated using Pajek64 v.3.14.

Genetic differentiation among localities were determined by non-hierarchical analyses of molecular variance (AMOVA) estimated using the software Arlequin v.3.5.1.2 [38], *D* statistic*s*, *G*_ST_ and their pairwise estimates [40], [41]. Also, hierarchical AMOVA’s were made for the sampling year and host plant. *D* statistics and *G*_ST_ were calculated with a R script provided by Pennings et al. [41]. Statistical significances were assessed with 1,000 permutations.

Because of the power to detect differentiation with *D* and *G*_ST_ is reduced when samples are small, DNA sequences were analyzed as if shorter fragments were sequenced as recommended by Pennings et al. [41].

### Discriminant analysis of principal components

Based on haplotype distribution and pairwise statistics, groups were establish and assured by discriminant analysis of principal components (DAPC) [42] with the R package adegenet v1.3–6. The genetic variability within and among groups was assessed by hierarchical AMOVA and associated F-statistics using the software Arlequin v.35.1.2 [38].

DAPC was determined by calculating the optimal number of components to be retained in the principal component analysis (PCA) by calculating the *a*-score. The *a*-score is given by the true assignment probability of individuals to their population (Pt) minus the assignment probability for individuals from randomly permuted populations (Pr) (100 permutations using the optim.*a*.score function in adegenet). We then determined the mean *a*-score (Pt-Pr) from 10,000 permutations for each group and calculated a *p*-value as the proportion of permutations with an *a*-score greater than 0.

### Demographic history

The demographic history of the sampled localities and groups were inferred based on the mismatch distribution analysis, which analyze the distribution of pairwise differences among sequences [43]. According to simulations, demographically stable or admixed populations must present a multimodal distribution, whereas populations that have experienced a recent expansion generally show a unimodal distribution [43]. The adjustment to the population expansion model was determined by the sum of the squared deviations (SSD) and the raggedness index (r), with significance evaluated by 1,000 permutations under the sudden expansion model. All analyzes were developed in the software Arlequin v.3.5.1.2 [38].

## Results

We amplified a 996 bp region of the COI gene for the 202 analyzed individuals and found 28 polymorphic sites, from which 19 were non-synonymous mutation sites. The average *p*-distance among sequences was 0.004 (range: 0.001–0.009), and 47 haplotypes (H) were identified (GeneBank Accession number: KC354739–KC354785). The molecular variability indices showed high haplotype diversity (H-mean  = 0.839; range: 0–1.00) and low nucleotide diversity (π-mean  = 0.002; range: 0–0.004) for the total sample.

Single haplotypes represented 70% (33/47) of all haplotypes, with the remaining 30% occurring in more than one locality (Table 1). Haplotype 1 (H1) was the most frequent, representing 34% (68/202) of the total sample, and widely distributed being found in 22 localities. H2 was the second most frequent haplotype representing 11% (23/202), and was found in 12 localities. H3 represented 7% (14/202) and was spread among 7 localities. The remaining frequent haplotypes were found in less than 6 localities (Table 1).

**Figure 1:**
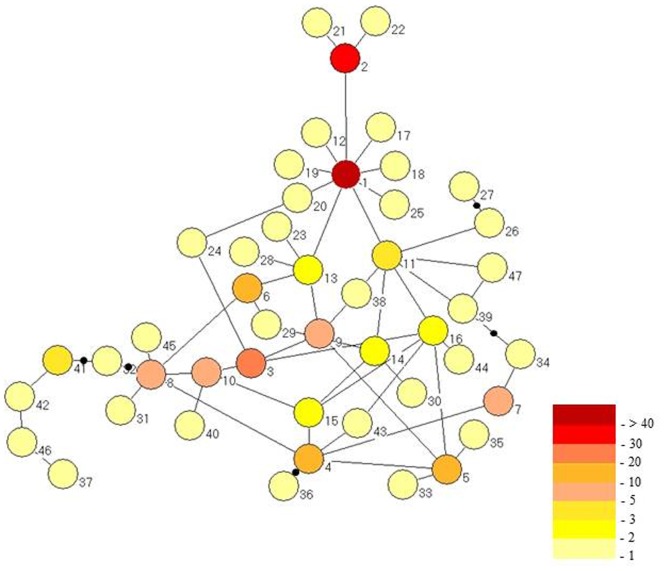
Haplotype network of populations of *Diaphorina citri* from Brazil based on partial sequences of the COI gene (996 bp), built by using the TCS program. Each circle represents a haplotype and circles are gradually colored depending on the frequency haplotypes were observed, from one occurrence (light yellow) to more than 40 occurrences (dark red).

The 47 mtDNA haplotypes were linked in a unique parsimony network (Fig. 1). Haplotype network topology shows a complex and intricate connection pattern. H1 is the hub of a “star-like” pattern. Satellite haplotypes are all one mutation step distant from H1, H2 being one of these. H3 is linked with frequent haplotypes and is separated by three mutational steps from H1.

Non-hierarchical AMOVA resulted in a high *F*_ST_ value (*F*_ST_  = 0.26, p<0.0001), which is indicative of genetic structure. The average *D* value among all samples was 0.52, highlighting the genetic structure among samples. The *G*_ST_ value (*G*_ST_  = 0.15) was the smallest of the three indices. However, *G*_ST_ is greatly underestimated when heterozygosity and variability is high [41] as in the populations of *D*. *citri* we analyzed.

The effect of fragment size on *D*, *G*_ST_ and *p-*values demonstrated that 500 bp were needed to obtain significant results (data not illustrated). *D*-power did not reduce with longer fragments, indicating the dataset has the required resolution to detect population structure.

**Figure 2:**
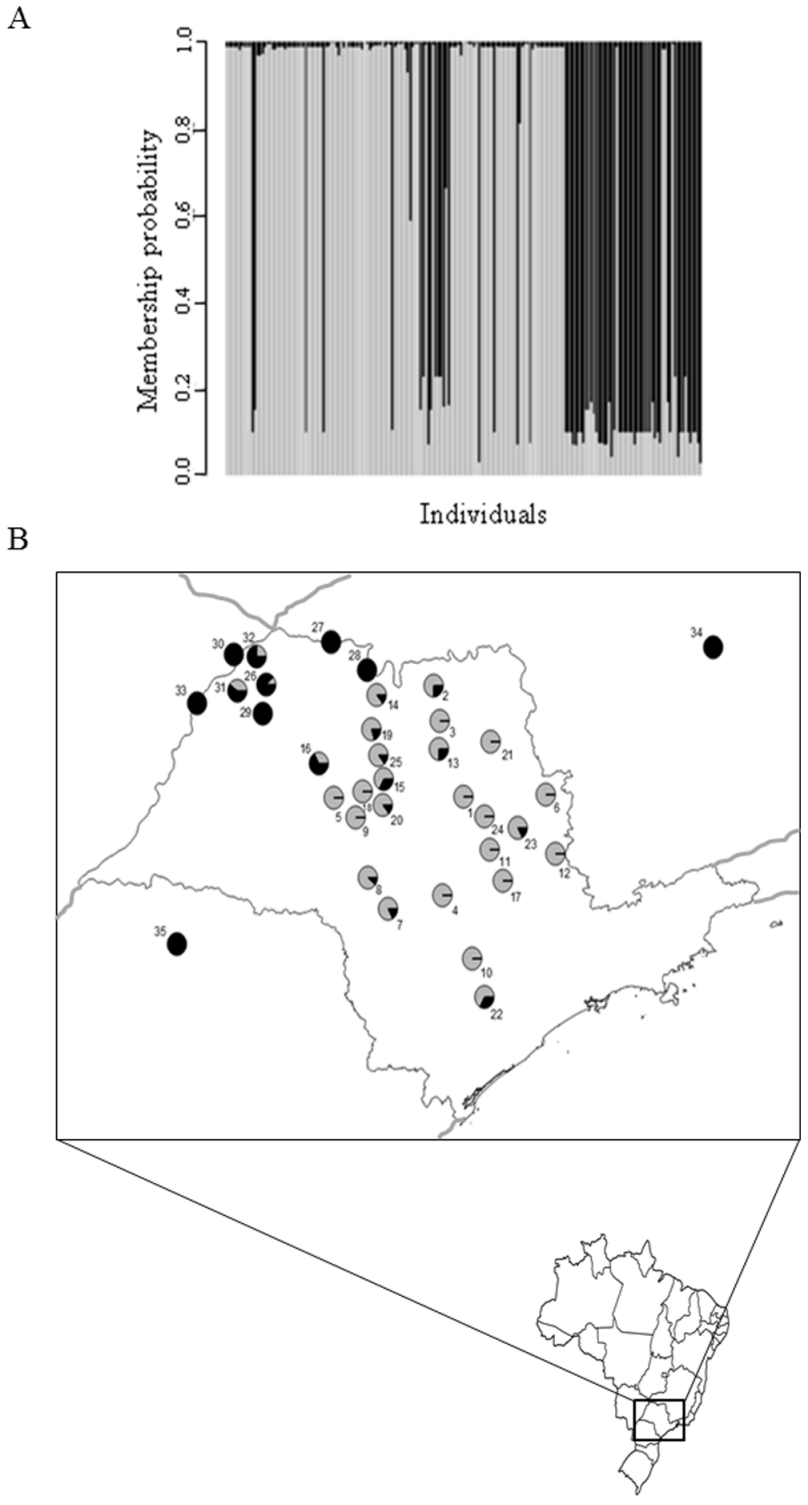
a) Membership probability of each individual to belong to group I or II, b) Membership probability plot on a map of the state of São Paulo. Group I in grey and Group II in black. Numbers on the map refer to the different localities sampled as reported in Table 1.

The presence of two regional groups genetically differentiated was confirmed by the DAPC (Fig. 2a). The probabilities of each individual belong to a group were plotted by locality on the map of the state of São Paulo (Fig. 2b), showing a geographic structure. The hierarchical AMOVA between these two groups showed an *F*_ST_  = 0.33, *p*<0.0001, demonstrating that Brazilian populations of *D. citri* are geographically structured (Table 2). Neither the sampling year nor the host plant yielded significant hierarchical AMOVAs, indicating these variables did not structure the genetic variability observed in the populations of *D. citri* we analyzed.

**Table 2:** Analysis of molecular variance (AMOVA) for *Diaphorina citri* samples using *COI* sequences.

Source of variation	*Df*	Sum of squares	Percentage of variation
**One group**			
Among populations	36	87.67	26.03
Within populations	164	137.52	73.97
		F_ST_: 0.26 P = 0.00	
**Two groups**			
Among groups	1	22.33	20.13
Among populations within groups	33	59.42	12.86
Within groups	152	135.77	68.01
		F_SC_: 0.16 P = 0.00	
		F_ST_: 0.33 P = 0.00	
		F_CT_: 0.20 P = 0.00	

A group denominated as Group I was formed by localities 1 to 25 (Table 1) and H1 was the most frequent haplotype in this group. The other group, Group II, is formed by localities 26 to 35 (Table 1), with H2 as the most frequent haplotype in this group.

**Figure 3:**
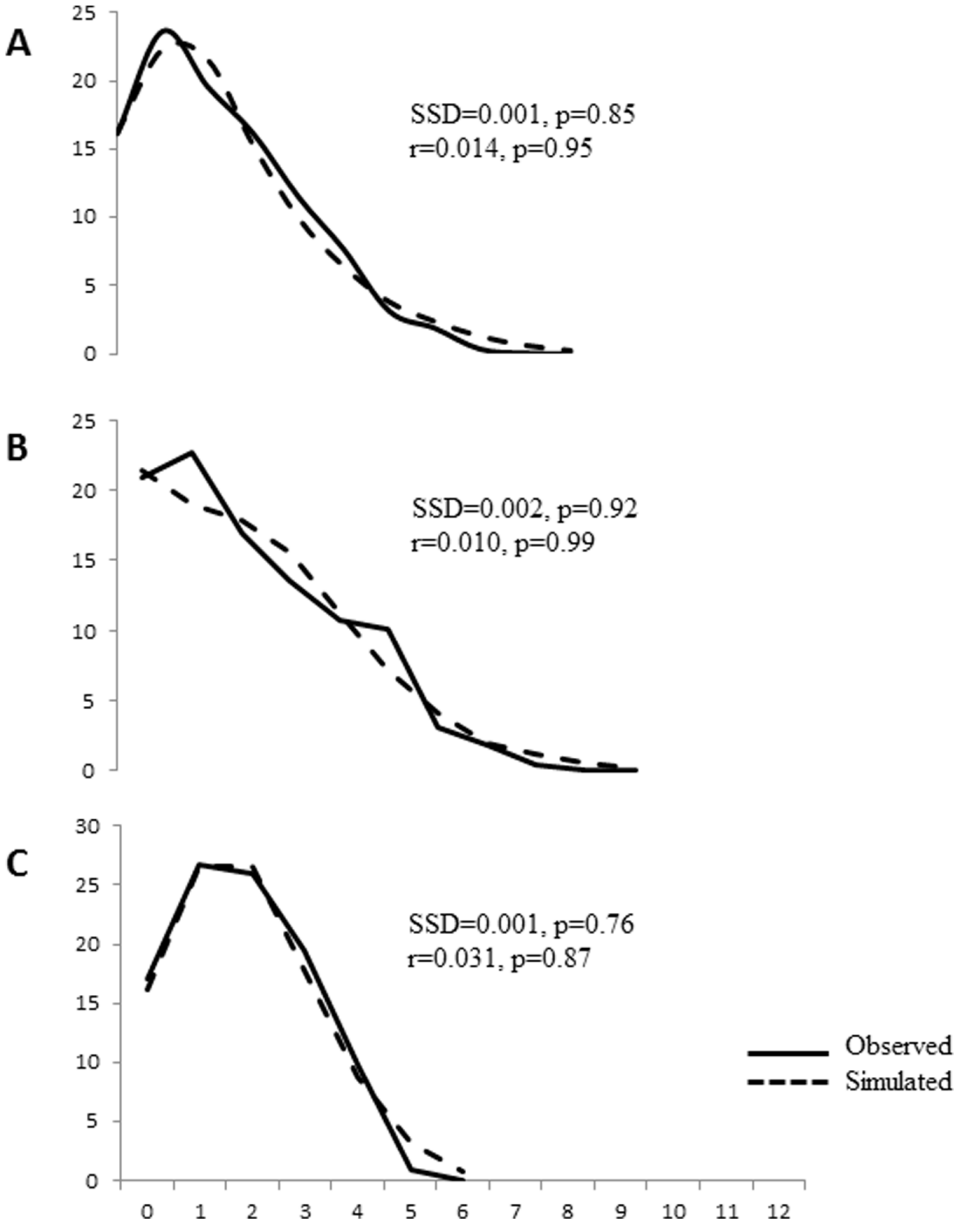
Mismatch curves of *Diaphorina citri* from the whole sample (a), and from group I (b) and II (c) independently.

The demographic history for both groups seems to be similar, as the mismatch distribution analyses resulted in a unimodal pattern for the whole sample and both groups (Fig. 3). This pattern is indicative of population expansion and could also be observed by the diversity indices obtained (Group I: π  = 0.002±0.001; H^  = 0.791±0.035; Group II: π  = 0.001±0.001; H^  = 0.829±0.046), which showed a high number of genetically closely related haplotypes. We also detected one non-synonymous mutation with striking differences in frequency between the two groups. A thymine is replaced by a cytosine at position 991 in this non-synonymous mutation, leading to a change from the amino acid phenylalanine to leucine. While thymine occurs in 86% of the sequences of Group I, cytosine is represented in 84.8% of the sequences of Group II. Thus, phenylalanine predominates in Group I and leucine in Group II.

## Discussion

The several approaches we used for the population genetic analysis of *D. citri* have all supported the geographic structure of the genetic diversity observed. We demonstrate here that the genetic diversity of *D. citri* is distributed in two groups: Group I (a group located at the eastern side of the state of São Paulo) and Group II (a group located at the western side of the state of São Paulo). The historical demography indicates that these populations expanded from an ancestral population with a small population size. Here, we argue about two possible directions of invasions, different reasons for genetic structuring and the high diversity found.

Two hypothetical scenarios of invasions were considered to explain the genetic variability distribution of *D. citri*. In the first scenario, invasion of São Paulo occurred at the eastern region of the state, and the dispersion process followed to the central-western regions, with a reduction in the frequency of the H1 haplotype as the populations further established in the western region.

Although detailed historical information of *D. citri* distribution in Brazil is missing, the first scenario is supported by the fact that the first report of this species in Brazil was made in Rio de Janeiro city (formely known as Guanabara) [32]. The states of São Paulo and Rio de Janeiro have a strong social and economic connection, therefore the transportation of agricultural products like citrus trees and fruits is very frequent, which may have facilitated the spread of *D. citri* from Rio de Janeiro to São Paulo.

However, data on spread of *D. citri* and on the history of citrus plants can also support a second proposed scenario. In the second scenario, invasion occurred from bordering states and then spread from the western region of São Paulo towards to the central and eastern regions. This second scenario is supported by the fact that populations sampled in the states of Paraná (southern border), Mato Grosso do Sul (western border) and Minas Gerais (northern border) are dominated by the H2 haplotype, like the other populations from the western border of São Paulo, which form Group II.

The history of citrus and the records of *D. citri* in Brazil also support the second scenario proposed. Citrus was introduced in Brazil around 1530 [44] and may have followed settlers/explorers to several regions of the country. This early movement would explain the detection of *D. citri* in the north (Amazônia, Pará), northeast (Bahia, Ceará, Pernambuco) and southeast (São Paulo) regions of Brazil 27 years after its first record by Costa Lima [45]. *D. citri* is reported to sustain flight activity for a short period [46] and to fly over short distances [47], [48], most likely due to the weak wing-associated musculature (as reviewed by [27]). Long-distance movement of *D. citri* has been indicated as possible [27], but long-distance movement has been suggested to require repeated short-distance flights [46]. Hence, is unlikely *D. citri* would have dispersed to regions of Brazil thousands of kilometres apart from each other in a short period of time after its first record, supporting our hypothesis that *D. citri* invaded Brazil much before it was first reported by Costa Lima [45].

The existence of a population solely represented by H1 in one of the most north-western states of Brazil, Roraima, is likely due to anthropic activities. Although data on the dispersion capabilities of *D. citri* is controversial (from 6 to 100 m), this species has a short dispersion capacity [46], [47], [48]. Yet, citrus has only recently been commercially produced in Roraima, and the population was collected in orange jasmine, an ornamental plant commercially produced in São Paulo and distributed throughout the country. Besides, sampling efforts to collect this species on citrus in this region has been unsuccessful [49].

There have been other efforts to understand the genetic diversity of *D. citri* in America [50] and worldwide [34] using the same molecular marker we have applied. However, these efforts used a shorter sequence, which may explain the reduced haplotype diversity they reported as compared with the one we described. De Leon et al. [50] identified 23 haplotypes from populations collected in several countries in America, while the worldwide haplotype diversity of *D. citri* reported by Boykin et al. [34] is even lower (only 8 haplotypes). Both of these studies included populations of *D. citri* sampled from the state of São Paulo, Brazil. Comparisons of the haplotypes of *D. citri* we detected with those reported from Brazil in these studies were only conducted with the haplotypes reported by Boykin et al. [34]. In this case, the most frequent haplotype we detected in São Paulo (H1) was not identified by Boykin et al. [34], meaning that their inferences on the genetic distribution scale of *D. citri*, when considering the Brazilian samples, may be misleading.

Genetic diversity of invasive populations is always considered low due to the reduced size of propagules and bottleneck events [37]. This statement contrasts with the high genetic diversity found here for this invasive pest. This scenario does not seem to be exclusively explained by mutation or divergence of subpopulations, as the nearly 75 years from the first record of *D. citri* in Brazil would be a short period to allow for such diversity. Multiple invasions would be an alternative explanation for the high genetic diversity observed.

Multiple invasions seem to be common in invasive processes, and genetically structured populations are highly unlikely in this scenario [51]. Nevertheless, Excoffier et al. [52] proposed that population structure may occur during the range shift process. The scenario for multiple invasions is favoured by the detection of the HLB disease in Brazil only in 2004 [24], despite the fact that *D. citri* has been known to occur since the late 1930 s [32]. If population structure arise during the range shift process [52] and multiple invasion events would support the introduction of the disease much after its vector was introduced in Brazil, one could argue that the less common H2 haplotype would represent a second, more recent event of invasion and be the source of the introduction of *Ca. Liberibacter* in the state of São Paulo. If this would have been the case, we should expect the early detection of infected plants and higher infection rates in groves in the northwest region, but the disease was first detected and infection rates are higher in the core region of the state where the H1 haplotype dominates. Nevertheless, the molecular data we have available up to this point does not allow for appropriate time estimations and the determination of the order of the multiple invasions events that may have occurred.

The geographic structure currently observed for *D. citri* in the state of São Paulo could be a result of a process of range expansion, as demonstrated in simulation studies by Excoffier et al. [52]. Another explanation is that the geographic distribution of the two major haplotypes (H1 and H2) may have been driven by selection on mitochondrial DNA, as *D*. *citri* populations were spread from one end of the state to the other.

Selection on mitochondrial DNA of *D. citri* was inferred by several non-synonymous mutations that were detected. The frequency of the non-synonymous mutation at position 991, which leads to an amino acid change, could be a result of selection and/or different demographic histories, as each group could also have originated from different invasion events. Although is not clear how mitochondrial DNA selection evolves [22], mutation of mitochondrial DNA has been directly linked with phenotypic selection [53], resulting in phenotypes expressing diverse fitness traits [53], [54]. The distribution of groups defined by H1 and H2 matches areas with different levels of HLB-symptomatic trees (southeast region  = 14.8% HLB-symptomatic trees; northwest region of the state of São Paulo  = 0.3% HLB-symptomatic trees) [55], suggesting that the disease would be much more common in the area dominated by the H1 haplotype (Group I) if compared to the H2 haplotype (Group II). Nevertheless, a number of other factors (presence of abandoned groves, citrus cultivars available, management strategies adopted, among others) have also been suggested to affect the distribution of the disease through the different regions of the state of São Paulo (Dr. Renato Bessanezi, Fundecitrus, Personal communication).

Selection of new phenotypes may be particularly worrisome as successful invasions may occur after severe bottleneck events or even with the invasion of a single mated female depending on its level of heterozygosity [56]. The Asian citrus psyllid is under intense selection pressure due to the massive use of pesticides as a management approach to avoid/reduce the spread of the HLB-causing agent [57], [58]. This selection pressure can eliminate haplotypes and favor the expansion of rare haplotypes with different fitness attributes, affecting pest and/or disease management strategies.

Our data on the genetic structure of *D. citri* in Brazil provide an optimistic scenario for HLB management in Brazil if the disease is contained within the state of São Paulo. The observation that *D. citri* populations are genetically structured indicates a low level of genetic material exchange among different populations depending on their group. Reduced genetic flow among groups that are geographically structured is another indication that this species has reduced dispersion capacity and, therefore, would be more amenable to containment strategies to reduce the spread of the disease. Nevertheless, the association of *D. citri* with orange jasmine and the free movement of these, as well as citrus seedlings, pose major risks for disease dissemination.

Genetic variability can be affected by a number of variables, such as the maternally-inherited secondary symbiont *Wolbachia* that can also drive host haplotype selection [59]. However, we have demonstrated earlier the genetic diversity of *D. citri* has not been affected by *Wolbachia* infection [60]. Use of additional markers (nuclear genes) or approaches such as microsatellites, RADSeq and Genotyping-by-sequencing (GBS) could improve the understanding of the population genetic structure and contribute to a better understanding of the processes of divergence in *D. citri*.

As a conclusion, our investigation on the intraspecific genetic variability of *D. citri* in Brazil led to the recognition of two geographic groups in the region of São Paulo and bordering states. We were able to demonstrate considerable genetic diversity that suggests multiple invasion events. We also argued that the COI of *D. citri* may be under non neutral selection; therefore that further assessment of the genetic variability of *D*. *citri* populations is needed in target areas for improved control.
